# Poisoning by Winslow’s Soothing Syrup

**Published:** 1868-09

**Authors:** 


*Poisoning by Winslow's Soothing Syrup.*—Dr. C——, of Washington, D. C., relates in the *Medical and Surgical Reporter*, a case of poisoning by the above nostrum, and the editors of that journal remark as follows: "We have no doubt that this is a clear case of poisoning, and the sale of Winslow's Soothing Syrup should be prohibited by law, as dangerous and destructive of life. We have known several instances where the child suffered severely from the use of it."

The examination of Dr. Charles P. Powers on a charge of causing the death of Mrs. Mary Abbie Bowen, by mal-practice on the 5th instant, in his office at No. 8 Boylton street, Boston, resulted in his being committed for trial in default of $10,000 bail.

